# Diethyl (1-hydr­oxy-1-phenyl­ethyl)phospho­nate

**DOI:** 10.1107/S1600536809011428

**Published:** 2009-03-31

**Authors:** M. Nawaz Tahir, Nurcan Acar, Hamza Yilmaz, Muhammad Ilyas Tariq, Ghulam Hussain

**Affiliations:** a Department of Physics, University of Sargodha, Sargodha, Pakistan; b Department of Chemistry, Faculty of Science, University of Ankara, Ankara, Turkey; c Department of Chemistry, University of Sargodha, Sargodha, Pakistan

## Abstract

The title compound, C_12_H_19_O_4_P, has a distorted tetra­hedral geometry around the P atom. The molecules form  dimers with *R*_2_^2^(10) ring motifs due to inter­molecular O—H⋯O hydrogen bonds. The double-bonded O atom of the phospho­nate group behaves as an acceptor and the hydr­oxy group acts as a donor. Both of the ethyl groups are disordered with occupancies of 0.55:0.45 and 0.725:0.275.

## Related literature

For phospho­nate compounds, see: Acar *et al.* (2009 ▶); Tahir *et al.* (2007 ▶, 2009 ▶). For related structures, see: deMendonca *et al.* (1996 ▶); Feng *et al.* (2007 ▶). For hydrogen-bond motifs, see: Bernstein *et al.* (1995 ▶).
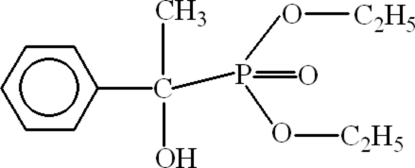


## Experimental

### 

#### Crystal data

C_12_H_19_O_4_P
*M* *_r_* = 258.24
Monoclinic, 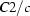

*a* = 20.1187 (12) Å
*b* = 8.4488 (14) Å
*c* = 18.4833 (12) Å
β = 116.991 (4)°
*V* = 2799.6 (5) Å^3^
*Z* = 8
Mo *K*α radiation
μ = 0.20 mm^−1^
*T* = 296 K
0.28 × 0.22 × 0.18 mm

#### Data collection

Enraf–Nonius CAD-4 diffractometer
Absorption correction: ψ scan (*MolEN*; Fair, 1990 ▶) *T*_min_ = 0.949, *T*_max_ = 0.969
2753 measured reflections
2664 independent reflections
1726 reflections with *I* > 2σ(*I*)
*R*_int_ = 0.011
3 standard reflections frequency: 120 min intensity decay: −1.2%

#### Refinement

*R*[*F* ^2^ > 2σ(*F* ^2^)] = 0.059
*wR*(*F* ^2^) = 0.197
*S* = 1.02
2664 reflections
162 parameters
6 restraints
H-atom parameters constrained
Δρ_max_ = 0.42 e Å^−3^
Δρ_min_ = −0.41 e Å^−3^

### 

Data collection: *CAD-4 EXPRESS* (Enraf–Nonius, 1993 ▶); cell refinement: *CAD-4 EXPRESS*; data reduction: *MolEN* (Fair, 1990 ▶); program(s) used to solve structure: *SHELXS86* (Sheldrick, 2008 ▶); program(s) used to refine structure: *SHELXL97* (Sheldrick, 2008 ▶); molecular graphics: *ORTEP-3 for Windows* (Farrugia, 1997 ▶) and *PLATON* (Spek, 2009 ▶); software used to prepare material for publication: *WinGX* (Farrugia, 1999 ▶).

## Supplementary Material

Crystal structure: contains datablocks global, I. DOI: 10.1107/S1600536809011428/bq2131sup1.cif

Structure factors: contains datablocks I. DOI: 10.1107/S1600536809011428/bq2131Isup2.hkl

Additional supplementary materials:  crystallographic information; 3D view; checkCIF report

## Figures and Tables

**Table 1:** Selected bond lengths (Å)

P1—O2	1.461 (3)
P1—O3	1.555 (3)
P1—O4	1.551 (3)
P1—C7	1.828 (3)
O1—C7	1.420 (4)

**Table 2:** Hydrogen-bond geometry (Å, °)

*D*—H⋯*A*	*D*—H	H⋯*A*	*D*⋯*A*	*D*—H⋯*A*
O1—H1⋯O2^i^	0.8200	1.9100	2.709 (4)	163.00

Symmetry code: (i) 
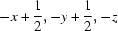
.

**Fig. 1.**
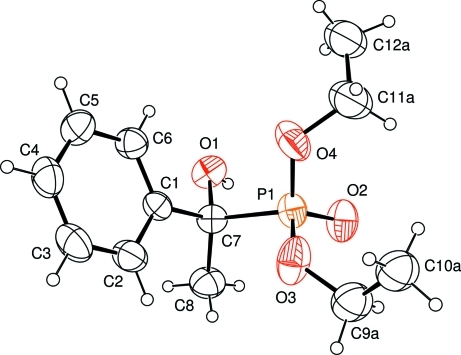
A view of (I), with the atom-numbering scheme and 30% probability displacement ellipsoids. Ethyl groups having higher occupancy ratios are selected.

**Fig. 2.**
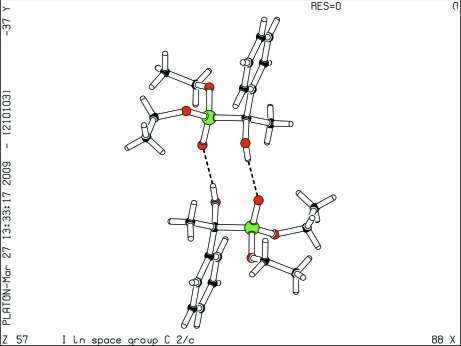
The figure (PLATON: Spek, 2009) which shows the formation of dimers through hydrogen bonding forming R22(10) motifs. Ethyl groups having higher occupancy ratios are selected.

## supplementary crystallographic information

### Comment

In continuation to the study of phosphonate compounds (Acar *et al.*, 2009; Tahir *et al.*, 2007, 2009), we, herein report the preparation and crystal structure of the title compound (I), (Fig. 1.).

The crystal structures of (II) Diethyl 1-hydroxy-1-(pyridin-2-yl)ethyl phosphonate (Feng *et al.*, 2007) and (III) Diethyl (1-hydroxy-1-methyl-3-phenyl- 2-propynyl)phosphonate (deMendonca *et al.*, 1996) has been reported, previously. The title compound (I) has distorted tetrahedral geometry around phosphorus atom (Table 1.) and differs from (II) as pyridin ring has been replaced by the phenyl ring. It is also dimerized (Fig. 2.) forming ring motifs *R*_2_^2^(10) (Bernstein *et al.*, 1995) due to intermolecular H-bonding of O–H···O type (Table 2.). Both of the ethyl groups are disordered having occupancy ratios of 0.55:0.45 and 0.725:0.275, respectively. There does not exist any kind of π-interaction.

### Refinement

H-atoms were positioned geometrically, with O-H = 0.82 Å for hydroxy, C-H = 0.93, 0.96 and 0.97 Å for aromatic, methyl and ethylene moieties and constrained to ride on their parent atoms, with U_iso_(H) = xU_eq_(C, O), where x = 1.5 for methyl H, and x = 1.2 for all other H atoms. The higher values of the refinement parameters and the thermal elipsoids indicated the disorder of ethyl groups. The disorder was resolved using DFIX and EADP commands.

### Crystal data

**Table:**

C_12_H_19_O_4_P	*F*(000) = 1104
*M**_r_* = 258.24	*D*_x_ = 1.225 Mg m^−^^3^
Monoclinic, *C*2/*c*	Melting point: 383 K
Hall symbol: -C 2yc	Mo *K*α radiation, λ = 0.71073 Å
*a* = 20.1187 (12) Å	Cell parameters from 25 reflections
*b* = 8.4488 (14) Å	θ = 10.2–18.1°
*c* = 18.4833 (12) Å	µ = 0.20 mm^−^^1^
β = 116.991 (4)°	*T* = 296 K
*V* = 2799.6 (5) Å^3^	Prismatic, colorless
*Z* = 8	0.28 × 0.22 × 0.18 mm

### Data collection

**Table:**

Enraf–Nonius CAD-4 diffractometer	*R*_int_ = 0.011
ω/2θ scans	θ_max_ = 25.7°, θ_min_ = 2.5°
Absorption correction: ψ scan (*MolEN*; Fair, 1990)	*h* = −21→24
*T*_min_ = 0.949, *T*_max_ = 0.969	*k* = −10→0
2753 measured reflections	*l* = −22→0
2664 independent reflections	3 standard reflections every 120 min
1726 reflections with *I* > 2σ(*I*)	intensity decay: −1.2%

### Refinement

**Table:**

Refinement on *F*^2^	Primary atom site location: structure-invariant direct methods
Least-squares matrix: full	Secondary atom site location: difference Fourier map
*R*[*F*^2^ > 2σ(*F*^2^)] = 0.059	H-atom parameters constrained
*wR*(*F*^2^) = 0.197	*w* = 1/[σ^2^(*F*_o_^2^) + (0.1174*P*)^2^ + 1.8226*P*] where *P* = (*F*_o_^2^ + 2*F*_c_^2^)/3
*S* = 1.02	(Δ/σ)_max_ < 0.001
2664 reflections	Δρ_max_ = 0.42 e Å^−^^3^
162 parameters	Δρ_min_ = −0.41 e Å^−^^3^
6 restraints	

### Special details

**Table:**

Experimental. The structure was solved by Patterson method using *SHELX86*. The whole molecule was recognized.
Geometry. Bond distances, angles *etc*. have been calculated using the rounded fractional coordinates. All su's are estimated from the variances of the (full) variance-covariance matrix. The cell e.s.d.'s are taken into account in the estimation of distances, angles and torsion angles

### Fractional atomic coordinates and isotropic or equivalent isotropic displacement parameters (Å^2^)

**Table:**

	*x*	*y*	*z*	*U*_iso_*/*U*_eq_	Occ. (<1)
P1	0.16206 (5)	0.43490 (11)	0.02880 (5)	0.0706 (3)	
O1	0.20371 (14)	0.1469 (3)	0.07797 (16)	0.0810 (9)	
O2	0.19607 (15)	0.4445 (3)	−0.02609 (15)	0.0956 (11)	
O3	0.1602 (2)	0.5940 (3)	0.07017 (18)	0.1071 (13)	
O4	0.07969 (14)	0.3787 (4)	−0.01170 (14)	0.1054 (12)	
C1	0.17069 (15)	0.2904 (3)	0.16722 (16)	0.0604 (9)	
C2	0.1870 (2)	0.3950 (5)	0.23087 (19)	0.0814 (11)	
C3	0.1507 (2)	0.3836 (6)	0.2789 (2)	0.0939 (15)	
C4	0.0982 (2)	0.2709 (5)	0.2640 (2)	0.0892 (15)	
C5	0.0807 (2)	0.1687 (5)	0.2010 (2)	0.0857 (14)	
C6	0.11694 (18)	0.1776 (4)	0.1532 (2)	0.0728 (11)	
C7	0.20991 (16)	0.2986 (3)	0.11360 (18)	0.0642 (10)	
C8	0.29101 (18)	0.3474 (5)	0.1597 (2)	0.0886 (14)	
C9A	0.1700 (12)	0.7452 (17)	0.0422 (8)	0.108 (2)	0.550
C10A	0.0980 (6)	0.8190 (12)	−0.0106 (7)	0.108 (2)	0.550
C11A	0.0250 (5)	0.4095 (9)	−0.0930 (4)	0.127 (2)	0.725
C12A	−0.0046 (4)	0.2635 (9)	−0.1369 (4)	0.127 (2)	0.725
C12B	−0.0142 (11)	0.364 (3)	−0.1501 (12)	0.127 (2)	0.275
C9B	0.1764 (16)	0.753 (2)	0.0580 (10)	0.108 (2)	0.450
C10B	0.1267 (7)	0.8175 (15)	−0.0212 (8)	0.108 (2)	0.450
C11B	0.0478 (10)	0.285 (3)	−0.0825 (10)	0.127 (2)	0.275
H2	0.22265	0.47350	0.24141	0.0978*	
H3	0.16250	0.45393	0.32172	0.1125*	
H4	0.07422	0.26333	0.29669	0.1071*	
H1	0.23442	0.13894	0.06047	0.0971*	
H8C	0.31674	0.27561	0.20397	0.1151*	
H9A	0.19892	0.73346	0.01251	0.1301*	0.550
H9B	0.19760	0.81335	0.08850	0.1301*	0.550
H10A	0.07397	0.76033	−0.06032	0.1410*	0.550
H10B	0.10608	0.92596	−0.02229	0.1410*	0.550
H10C	0.06693	0.81905	0.01637	0.1410*	0.550
H11A	0.04719	0.47110	−0.12064	0.1519*	0.725
H11B	−0.01536	0.47112	−0.09211	0.1519*	0.725
H12A	−0.02537	0.28317	−0.19414	0.1646*	0.725
H12B	−0.04285	0.22371	−0.12424	0.1646*	0.725
H12C	0.03471	0.18690	−0.12131	0.1646*	0.725
H5	0.04399	0.09245	0.19003	0.1028*	
H6	0.10484	0.10608	0.11079	0.0872*	
H8A	0.29401	0.45276	0.18045	0.1151*	
H8B	0.31367	0.34490	0.12383	0.1151*	
H9C	0.22718	0.75826	0.06495	0.1301*	0.450
H9D	0.17378	0.81967	0.09945	0.1301*	0.450
H10D	0.07590	0.79776	−0.03240	0.1410*	0.450
H10E	0.13659	0.76753	−0.06191	0.1410*	0.450
H10F	0.13462	0.92943	−0.02149	0.1410*	0.450
H11C	0.03010	0.18697	−0.07032	0.1519*	0.275
H11D	0.08597	0.25945	−0.09889	0.1519*	0.275
H12D	−0.00459	0.36632	−0.19639	0.1646*	0.275
H12E	−0.01932	0.46968	−0.13469	0.1646*	0.275
H12F	−0.05948	0.30603	−0.16350	0.1646*	0.276

### Atomic displacement parameters (Å^2^)

**Table:**

	*U*^11^	*U*^22^	*U*^33^	*U*^12^	*U*^13^	*U*^23^
P1	0.0724 (5)	0.0812 (6)	0.0660 (5)	0.0143 (4)	0.0382 (4)	0.0060 (4)
O1	0.0893 (16)	0.0692 (14)	0.1046 (17)	0.0017 (12)	0.0616 (14)	−0.0126 (13)
O2	0.1085 (19)	0.109 (2)	0.0973 (17)	0.0255 (15)	0.0711 (16)	0.0183 (15)
O3	0.170 (3)	0.0692 (16)	0.1128 (19)	0.0340 (16)	0.091 (2)	0.0190 (14)
O4	0.0682 (15)	0.174 (3)	0.0634 (13)	0.0083 (16)	0.0207 (11)	0.0125 (16)
C1	0.0563 (15)	0.0638 (18)	0.0544 (14)	0.0051 (13)	0.0193 (12)	0.0006 (13)
C2	0.080 (2)	0.088 (2)	0.0687 (19)	−0.0086 (18)	0.0271 (17)	−0.0128 (17)
C3	0.101 (3)	0.119 (3)	0.0617 (19)	−0.001 (2)	0.0370 (19)	−0.020 (2)
C4	0.093 (3)	0.117 (3)	0.0685 (19)	0.005 (2)	0.0463 (19)	0.006 (2)
C5	0.082 (2)	0.093 (3)	0.091 (2)	−0.0069 (19)	0.047 (2)	0.004 (2)
C6	0.074 (2)	0.074 (2)	0.0723 (18)	−0.0027 (16)	0.0350 (16)	−0.0083 (16)
C7	0.0605 (16)	0.0632 (18)	0.0701 (17)	0.0023 (14)	0.0308 (14)	−0.0045 (15)
C8	0.0603 (19)	0.098 (3)	0.098 (2)	−0.0004 (18)	0.0275 (18)	0.001 (2)
C9A	0.114 (4)	0.088 (2)	0.117 (4)	−0.001 (2)	0.048 (3)	−0.003 (2)
C10A	0.114 (4)	0.088 (2)	0.117 (4)	−0.001 (2)	0.048 (3)	−0.003 (2)
C11A	0.118 (4)	0.119 (4)	0.105 (3)	−0.008 (3)	0.017 (3)	−0.009 (3)
C12A	0.118 (4)	0.119 (4)	0.105 (3)	−0.008 (3)	0.017 (3)	−0.009 (3)
C12B	0.118 (4)	0.119 (4)	0.105 (3)	−0.008 (3)	0.017 (3)	−0.009 (3)
C9B	0.114 (4)	0.088 (2)	0.117 (4)	−0.001 (2)	0.048 (3)	−0.003 (2)
C10B	0.114 (4)	0.088 (2)	0.117 (4)	−0.001 (2)	0.048 (3)	−0.003 (2)
C11B	0.118 (4)	0.119 (4)	0.105 (3)	−0.008 (3)	0.017 (3)	−0.009 (3)

### Geometric parameters (Å, °)

**Table:**

P1—O2	1.461 (3)	C5—H5	0.9300
P1—O3	1.555 (3)	C6—H6	0.9300
P1—O4	1.551 (3)	C8—H8A	0.9600
P1—C7	1.828 (3)	C8—H8B	0.9600
O1—C7	1.420 (4)	C8—H8C	0.9600
O3—C9A	1.425 (16)	C9A—H9A	0.9700
O3—C9B	1.424 (19)	C9A—H9B	0.9700
O4—C11A	1.427 (7)	C9B—H9C	0.9700
O4—C11B	1.41 (2)	C9B—H9D	0.9700
O1—H1	0.8200	C10A—H10A	0.9600
C1—C2	1.387 (4)	C10A—H10C	0.9600
C1—C6	1.375 (5)	C10A—H10B	0.9600
C1—C7	1.523 (5)	C10B—H10E	0.9600
C2—C3	1.385 (6)	C10B—H10D	0.9600
C3—C4	1.354 (6)	C10B—H10F	0.9600
C4—C5	1.361 (5)	C11A—H11A	0.9700
C5—C6	1.379 (6)	C11A—H11B	0.9700
C7—C8	1.515 (5)	C11B—H11C	0.9700
C9A—C10A	1.47 (2)	C11B—H11D	0.9700
C9B—C10B	1.45 (2)	C12A—H12A	0.9600
C11A—C12A	1.448 (11)	C12A—H12B	0.9600
C11B—C12B	1.47 (3)	C12A—H12C	0.9600
C2—H2	0.9300	C12B—H12D	0.9600
C3—H3	0.9300	C12B—H12E	0.9600
C4—H4	0.9300		
			
P1···H10E	3.1900	C8···H2	2.6800
O1···O2	3.127 (4)	C12B···H12D^ix^	3.0300
O1···O4	3.009 (4)	H1···O2^ii^	1.9100
O1···C9B^i^	3.366 (17)	H1···H10F^i^	2.5900
O1···O2^ii^	2.709 (4)	H1···H8B	2.2900
O1···C11B	3.399 (19)	H2···C8	2.6800
O1···C10B^i^	3.306 (13)	H2···H8A	2.2000
O2···O1^ii^	2.709 (4)	H3···O2^viii^	2.7200
O2···O1	3.127 (4)	H4···H10E^viii^	2.3400
O3···C2	3.238 (5)	H6···O1	2.3500
O4···C6	3.266 (4)	H8A···C2	2.7500
O4···O1	3.009 (4)	H8A···O3	2.8000
O1···H10F^i^	2.5200	H8A···C5^vii^	3.0800
O1···H9B^i^	2.8300	H8A···H2	2.2000
O1···H10B^i^	2.7300	H8B···O2	2.8300
O1···H9D^i^	2.8900	H8B···H11D^ii^	2.4300
O1···H6	2.3500	H8B···H1	2.2900
O2···H9A	2.5400	H8C···C2	3.0400
O2···H3^iii^	2.7200	H9A···H9A^x^	2.3200
O2···H1^ii^	1.9100	H9A···O2	2.5400
O2···H8B	2.8300	H9B···O1^vi^	2.8300
O2···H11A	2.7100	H9B···C3^vii^	2.9800
O2···H11D	2.5400	H9C···C3^vii^	3.0100
O3···H8A	2.8000	H9D···O1^vi^	2.8900
C2···O3	3.238 (5)	H10A···C5^iv^	3.0800
C3···C12B^iv^	3.44 (2)	H10B···O1^vi^	2.7300
C3···C9B^v^	3.59 (2)	H10B···H11C^vi^	2.6000
C6···O4	3.266 (4)	H10C···H11C^iv^	2.5600
C9B···O1^vi^	3.366 (17)	H10E···C4^iii^	2.9700
C9B···C3^vii^	3.59 (2)	H10E···P1	3.1900
C10B···O1^vi^	3.306 (13)	H10E···H4^iii^	2.3400
C11B···O1	3.399 (19)	H10F···H1^vi^	2.5900
C12B···C3^iv^	3.44 (2)	H10F···O1^vi^	2.5200
C2···H8A	2.7500	H11A···O2	2.7100
C2···H8C	3.0400	H11C···H10B^i^	2.6000
C3···H12E^iv^	3.0400	H11C···H10C^iv^	2.5600
C3···H9B^v^	2.9800	H11D···O2	2.5400
C3···H9C^v^	3.0100	H11D···H8B^ii^	2.4300
C4···H12E^iv^	3.1000	H12D···C12B^ix^	3.0300
C4···H10E^viii^	2.9700	H12D···H12D^ix^	2.0700
C5···H10A^iv^	3.0800	H12E···C3^iv^	3.0400
C5···H8A^v^	3.0800	H12E···C4^iv^	3.1000
			
O2—P1—O3	114.63 (18)	H8A—C8—H8C	109.00
O2—P1—O4	114.61 (15)	H8B—C8—H8C	109.00
O2—P1—C7	113.59 (16)	O3—C9A—H9A	109.00
O3—P1—O4	104.1 (2)	O3—C9A—H9B	109.00
O3—P1—C7	104.11 (14)	C10A—C9A—H9A	109.00
O4—P1—C7	104.63 (16)	C10A—C9A—H9B	109.00
P1—O3—C9A	123.9 (7)	H9A—C9A—H9B	108.00
P1—O3—C9B	132.8 (10)	O3—C9B—H9D	109.00
P1—O4—C11A	126.6 (4)	C10B—C9B—H9C	109.00
P1—O4—C11B	123.8 (9)	O3—C9B—H9C	109.00
C7—O1—H1	109.00	H9C—C9B—H9D	107.00
C2—C1—C7	122.0 (3)	C10B—C9B—H9D	109.00
C6—C1—C7	120.3 (3)	C9A—C10A—H10B	109.00
C2—C1—C6	117.7 (3)	C9A—C10A—H10A	109.00
C1—C2—C3	120.6 (4)	C9A—C10A—H10C	109.00
C2—C3—C4	120.5 (4)	H10A—C10A—H10B	110.00
C3—C4—C5	119.7 (4)	H10A—C10A—H10C	109.00
C4—C5—C6	120.4 (4)	H10B—C10A—H10C	109.00
C1—C6—C5	121.1 (3)	C9B—C10B—H10E	109.00
P1—C7—C1	111.1 (2)	C9B—C10B—H10F	110.00
P1—C7—C8	108.9 (2)	C9B—C10B—H10D	109.00
P1—C7—O1	105.7 (2)	H10D—C10B—H10E	109.00
O1—C7—C8	110.6 (3)	H10D—C10B—H10F	109.00
C1—C7—C8	113.0 (3)	H10E—C10B—H10F	110.00
O1—C7—C1	107.4 (2)	O4—C11A—H11A	109.00
O3—C9A—C10A	111.4 (16)	O4—C11A—H11B	109.00
O3—C9B—C10B	114.3 (16)	C12A—C11A—H11A	109.00
O4—C11A—C12A	111.1 (6)	C12A—C11A—H11B	109.00
O4—C11B—C12B	112.8 (19)	H11A—C11A—H11B	108.00
C1—C2—H2	120.00	O4—C11B—H11C	109.00
C3—C2—H2	120.00	C12B—C11B—H11D	109.00
C2—C3—H3	120.00	O4—C11B—H11D	109.00
C4—C3—H3	120.00	C12B—C11B—H11C	109.00
C3—C4—H4	120.00	H11C—C11B—H11D	108.00
C5—C4—H4	120.00	C11A—C12A—H12B	109.00
C4—C5—H5	120.00	C11A—C12A—H12C	109.00
C6—C5—H5	120.00	C11A—C12A—H12A	109.00
C1—C6—H6	119.00	H12A—C12A—H12C	110.00
C5—C6—H6	119.00	H12B—C12A—H12C	109.00
C7—C8—H8A	109.00	H12A—C12A—H12B	109.00
C7—C8—H8B	109.00	C11B—C12B—H12D	109.00
C7—C8—H8C	110.00	C11B—C12B—H12E	110.00
H8A—C8—H8B	109.00	H12D—C12B—H12E	110.00
			
O2—P1—O3—C9A	17.3 (11)	P1—O4—C11A—C12A	121.8 (6)
O4—P1—O3—C9A	−108.6 (11)	C6—C1—C2—C3	0.9 (5)
C7—P1—O3—C9A	142.0 (11)	C7—C1—C2—C3	−179.5 (3)
O2—P1—O4—C11A	−33.3 (5)	C2—C1—C6—C5	−0.2 (5)
O3—P1—O4—C11A	92.7 (5)	C7—C1—C6—C5	−179.8 (3)
C7—P1—O4—C11A	−158.4 (5)	C2—C1—C7—P1	−87.5 (3)
O2—P1—C7—O1	−62.4 (3)	C2—C1—C7—O1	157.5 (3)
O2—P1—C7—C1	−178.45 (19)	C2—C1—C7—C8	35.3 (4)
O2—P1—C7—C8	56.5 (3)	C6—C1—C7—P1	92.2 (3)
O3—P1—C7—O1	172.3 (3)	C6—C1—C7—O1	−22.9 (4)
O3—P1—C7—C1	56.2 (3)	C6—C1—C7—C8	−145.1 (3)
O3—P1—C7—C8	−68.9 (3)	C1—C2—C3—C4	−0.6 (6)
O4—P1—C7—O1	63.4 (3)	C2—C3—C4—C5	−0.4 (6)
O4—P1—C7—C1	−52.8 (2)	C3—C4—C5—C6	1.1 (6)
O4—P1—C7—C8	−177.8 (2)	C4—C5—C6—C1	−0.8 (6)
P1—O3—C9A—C10A	95.4 (14)		

Symmetry codes: (i) *x*, *y*−1, *z*; (ii) −*x*+1/2, −*y*+1/2, −*z*; (iii) *x*, −*y*+1, *z*−1/2; (iv) −*x*, −*y*+1, −*z*; (v) −*x*+1/2, *y*−1/2, −*z*+1/2; (vi) *x*, *y*+1, *z*; (vii) −*x*+1/2, *y*+1/2, −*z*+1/2; (viii) *x*, −*y*+1, *z*+1/2; (ix) −*x*, *y*, −*z*−1/2; (x) −*x*+1/2, −*y*+3/2, −*z*.

### Hydrogen-bond geometry (Å, °)

**Table:**

*D*—H···*A*	*D*—H	H···*A*	*D*···*A*	*D*—H···*A*
O1—H1···O2^ii^	0.8200	1.9100	2.709 (4)	163.00

Symmetry codes: (ii) −*x*+1/2, −*y*+1/2, −*z*.
